# Differential Effects of Assisted Reproduction Technology on Placental Epigenetics and Angiogenesis: Insights from Fresh, Frozen, and Egg Donation Pregnancies

**DOI:** 10.3390/life15121882

**Published:** 2025-12-10

**Authors:** Anna Maria Nuzzo, Stefano Canosa, Laura Moretti, Claudia Borbon, Marta Sestero, Bernadette Evangelisti, Alberto Revelli, Alessandro Rolfo

**Affiliations:** 1 Department of Surgical Sciences, Gynecology and Obstetrics 2, City of Health and Science-S. Anna University Hospital, University of Turin, 10126 Turin, Italy; l.moretti@unito.it (L.M.); cla.borbon@gmail.com (C.B.); alberto.revelli@unito.it (A.R.); alessandro.rolfo@unito.it (A.R.); 2 Obstetrics and Gynecology 1U, Physiopathology of Reproduction and IVF Unit, Department of Surgical Sciences, S. Anna Hospital, Università degli Studi di Torino, 10126 Torino, Italy; s.canosa88@gmail.com (S.C.); sesteromarta@gmail.com (M.S.); betty.evangelisti@gmail.com (B.E.)

**Keywords:** placenta, DNA methylation, angiogenesis, Assisted Reproductive Technology (ART), In Vitro Fertilization (IVF), fresh embryo transfer (ET), frozen-thawed embryo transfer (FET), egg donation (ED)

## Abstract

**Background:** The placenta plays a fundamental role in supporting fetal development, with angiogenesis being crucial for establishing an efficient maternal–fetal interface. Epigenetic mechanisms, particularly DNA methylation, can regulate the expression of angiogenesis-related genes and may be influenced by Assisted Reproductive Technology (ART), including In Vitro Fertilization (IVF) with fresh or frozen-thawed embryo transfer (ET and FET, respectively) and egg donation (ED), all potentially affecting placental vascular development and pregnancy outcomes. The present study compared global DNA methylation levels and the expression of Vascular Endothelial Growth Factor (VEGF), Placental Growth Factor (PlGF), and Soluble Fms-Like Tyrosine kinase-1 (sFlt-1) in placentae from physiological pregnancies obtained using ART versus those spontaneously conceived. **Methods:** Placental biopsies were collected from 98 physiological singleton term pregnancies (CTRL n = 29, ET n = 23, FET n = 25, ED n = 21). Global DNA methylation (5-mC) was quantified by ELISA Easy Kit; VEGF, PlGF, sFlt-1 mRNA and protein levels were assessed by Real-Time PCR and ELISA, respectively. **Results:** Global DNA methylation was significantly increased in FET and ED placentae compared with CTRL and ET. PlGF mRNA expression was upregulated in all ART groups, although protein levels were elevated only in ED placentae compared to CTRL and ET groups. VEGF mRNA was increased in FET placentae compared to CTRL, while protein levels showed a non-significant upward trend across ART groups. No differences in sFlt-1 expression were observed. Clinically, ART pregnancies were associated with significantly lower birth weight compared to CTRL, though values remained within the physiological range, and placental efficiency was preserved. **Conclusions:** Hypermethylation in FET and ED placentae may act as an epigenetic “buffer,” stabilizing vulnerable genomic regions and supporting the expression of pro-angiogenic factors. This adaptive mechanism likely helps to preserve placental function and fetal viability despite ART-related stressors, thereby mitigating the potential impact on birth weight.

## 1. Introduction

The placenta plays a vital role in supporting fetal development during pregnancy through nutrient exchange, gas transfer, and endocrine signaling. One of the most critical processes in early placentation is angiogenesis, which ensures an efficient fetal-maternal interface [1]. Fetal-placental angiogenesis initiates as early as day 21 post-conception and progresses throughout the first trimester, ending in a complex network of villous capillaries essential for placental function and fetal growth [2]. The formation and maintenance of the placental vascular network is tightly regulated by a dynamic balance between pro- and anti-angiogenic factors. Among the pro-angiogenic substances, Vascular Endothelial Growth Factor (VEGF) and Placental Growth Factor (PlGF) play a central role in promoting endothelial cell proliferation, migration, and new vessel formation. VEGF promotes “branching” angiogenesis, thus increasing vessel number and exchange surface, while PlGF mainly increases vessel caliber. In contrast, the soluble form of Fms-like Tyrosine Kinase-1 (sFlt-1) acts as an anti-angiogenic factor by sequestering VEGF and PlGF, preventing their interaction with endothelial receptors and inhibiting downstream biological activity [3,4]. An imbalance in this regulatory system, characterized by elevated circulating sFlt-1 levels, decreases PlGF concentration, and the resulting aberrantly low sFlt-1/PlGF ratio results in a systemic anti-angiogenic state. This dysregulation has been strongly associated with several serious pregnancy complications, including Preeclampsia (PE) and Fetal Growth Restriction (FGR) [5,6,7,8].

Emerging evidences suggest that epigenetic modifications, particularly DNA methylation, act as a regulatory framework, modulating the expression of angiogenesis-related genes and influencing the overall placental vascular development. In early gestation, the placenta is characterized by an overall low methylation level, which allows flexible gene regulation during a period of rapid tissue growth and extensive vascular remodeling [9,10,11]. DNA methylation typically occurs at cytosine residues within CpG dinucleotides of gene promoter regions, and is associated with transcriptional repression. The addition of methyl groups to DNA can hinder the binding of transcription factors or recruit methyl-binding proteins that promote chromatin condensation, making the DNA less accessible for transcription. This condensed chromatin state (heterochromatin) results in reduced gene expression [12,13]. As previously mentioned, the dysregulation of methylation patterns can alter the expression of angiogenesis-related genes and compromise placental vascularization, thereby contributing to pathological conditions as PE and FGR [14,15,16,17].

Altered DNA methylation profiles have been observed in gametes, embryos, cord blood, placenta, and offspring of pregnancies conceived by In Vitro Fertilization (IVF); this has been supposed to be linked to IVF procedures like gamete and embryo manipulation, embryo culture conditions, and exposure to a non-physiological hormonal environment. In theory, even other additional stressors, such as variations in temperature, pH, and oxygen tension that might occur during IVF, could disturb the epigenetic landscape during critical windows of early embryonic development [18,19,20,21,22]. Furthermore, the use of donor oocytes introduces another layer of complexity by separating the oocyte genetic from the epigenetic contribution of the uterine environment.

In egg donation (ED) cycles, embryos are generated from donated oocytes and are cryopreserved and subsequently thawed prior to transfer, thereby combining two potential sources of epigenetic perturbation: the intrinsic epigenetic status of the donor gamete and the stress associated with embryo freezing/thawing procedures. While Egg Donation (ED) can improve outcomes for individuals with poor oocyte quality, it may also influence epigenetic reprogramming and placental angiogenic gene expression. Moreover, the choice of embryo transfer strategy, specifically with Fresh (ET) or Frozen-Thawed (FET) embryos, may further modulate epigenetic patterns, including DNA methylation. In fresh ET, performed in the same cycle during which specific hormones are administered to obtain Controlled Ovarian Stimulation (COS), the endometrium is exposed to supraphysiological estrogenic levels, which may disrupt embryo-endometrial synchrony and interfere with the establishment of normal epigenetic marks. In contrast, FET is typically performed in a natural or hormonally regulated cycle, providing a more physiological endometrial environment that may reduce epigenetic disturbances, helping to explain the more favorable perinatal outcomes associated with FET [4,23].

The specific impact of IVF on DNA methylation remains an area of active investigation. Together, several factors related to IVF may influence DNA methylation and the expression of angiogenesis-related genes, thus altering placental vascular development and potentially affecting pregnancy outcomes. Understanding the epigenetic consequences of IVF is critical for optimizing treatment protocols and improving long-term health outcomes of IVF-conceived pregnancies. In the present study, we analyzed global DNA methylation patterns in placentae from fresh ET, FET, and ED IVF pregnancies, comparing them to those found in placentae from spontaneously conceived pregnancies. Moreover, we assessed the expression of placental pro-angiogenic PlGF, VEGF, and anti-angiogenic sFlt-1 in order to investigate whether epigenetic alterations associated with different IVF protocols are associated with changes in placental vascular signaling pathways.

## 2. Materials and Methods

### 2.1. Ethics Statement

The study was conducted at the Department of Surgical Sciences—Gynecology and Obstetrics Unit U2, Città della Salute e della Scienza—Sant Anna University Hospital, University of Turin (Turin, Italy). The study was performed in adherence to the Declaration of Helsinki. Informed consent allowing the collection and study of placenta samples was obtained in accordance with the ethics guidelines of the local Ethics Committee (approval protocol 0053289/CS2/1218, 24 May 2019).

### 2.2. Study Population and Tissue Collection

This study was conducted on 98 pregnant patients attending our university hospital between January 2019 and December 2020. They were divided into four sub-groups: spontaneous singleton pregnancies (controls, CTRL-group; n = 29); homologous IVF pregnancies resulting from fresh single blastocyst transfer (ET-group; n = 23); homologous IVF pregnancies resulting from vitrified-thawed single blastocyst transfer (FET-group; n = 25), and IVF pregnancies achieved through egg donation using vitrified-thawed blastocyst transfer (ED-group; n = 21).

The control group consisted of women with a healthy, uncomplicated singleton pregnancy, delivering at term, who showed no clinical or ultrasound signs of maternal, placental, or fetal disease. Additional exclusion criteria for this group included pre-existing chronic conditions (hypertension, diabetes, autoimmune disease), gestational complications (preeclampsia, gestational diabetes, fetal growth restriction), multiple pregnancy, smoking during pregnancy, and conception through any form of assisted reproduction. Patients undergoing IVF with autologous oocytes were selected according to strict ovarian reserve and metabolic criteria to reduce confounding biological variability. Inclusion criteria were: normal pre-treatment Body Mass Index (BMI 18–25), day 3 serum Follicle-Stimulating Hormone (FSH) < 12 IU/L, serum Anti-Müllerian Hormone (AMH) > 1.2 ng/mL, antral follicle count (AFC) > 8, regular menstrual cycles, no history of recurrent miscarriage, no uterine malformations or intrauterine pathologies and no chronic maternal disease. Controlled Ovarian Stimulation (COS), transvaginal ultrasound-guided oocyte retrieval, oocyte processing, Intra-Cytoplasmic Sperm Injection (ICSI), and embryo culture were performed according to our clinical practice, as described elsewhere [24]. Embryos were cultured using the Geri plus^®^ TLS (Genea Biomed, Sydney, Australia) with an integrated embryo monitoring system in microwells (one zygote/microwell) [25].

Embryos were cultured in pre-equilibrated Cleavage medium (Cook, Limerick, Ireland) overlaid with mineral oil up to day 3; at this stage, a change in medium was performed, and a new one (Blastocyst medium, Cook, Ireland) was added and kept until the blastocyst stage, when live embryos were assessed for transfer or cryopreservation.

Fresh transfer (ET-group) was performed on day 5 after ranking the available blastocysts on the basis of morphological criteria [26]; surplus good quality embryos were vitrified with Cryotop carrier using the Vitrification Kit (Kitazato, Tokyo, Japan) on day 5 or 6. Vitrified-thawed blastocyst transfer (FET-group) was performed on a modified natural cycle 7 days after ovulation trigger by hCG (10,000 IU subcutaneously) or 6 days after the detection of urinary LH peak, when the endometrial thickness reached at least 7 mm with a trilaminar (type 1) aspect.

In the ED group, vitrified-thawed blastocysts were derived from donated vitrified oocytes from healthy young donors fertilized with partner sperm and subsequently transferred as vitrified-thawed single blastocysts. Women were screened for the same maternal medical exclusion criteria as the homologous IVF groups. The transfer was performed using the same criteria as FET. This strategy, therefore, combines two potential modulators of epigenetic programming: the use of donor gametes and embryo vitrification/thawing, introducing an additional level of biological and epigenetic variability compared to homologous IVF cycles. In all cases, transvaginal progesterone (600 mg/day) was administered until the day of pregnancy test, performed 11 days after transfer, and continued until week 9 of gestational age in case of pregnancy. Embryo quality at the time of transfer was evaluated according to standard morphological scoring and the Istanbul consensus criteria for blastocyst assessment [26], ensuring comparability among ET, FET, and ED groups. Blastocyst morphology was graded by the embryologist using a two-digit scoring system, reflecting both the degree of blastocyst expansion and hatching stage (first digit) and the quality of the inner cell mass (ICM) and trophectoderm (TE) (second digit). Blastocysts were classified as excellent (11), good (12 or 21), average (22, 13, or 31), or poor (33, 23, or 32), with higher scores indicating suboptimal cellular development. Only embryos of appropriate and comparable quality were selected for transfer in all groups, minimizing potential bias related to embryo quality. Clinical pregnancy was confirmed by ultrasound visualization of a gestational sac with fetal heartbeat, performed 3 weeks after the positive pregnancy test.

All pregnant women were monitored in the second trimester with uterine Doppler flow velocimetry and in the third trimester by umbilical Doppler to evaluate in vivo placental development and vascularization. Umbilical artery Doppler waveforms were analyzed using the Pulsatility Index (PI), defined as peak systolic flow minus end diastolic flow, divided by mean flow. Normal PI values were defined according to gestational age-adjusted data.

Full-thickness tissue biopsies from case and control placentae were randomly collected, after spontaneous delivery or cesarean section, from the intermediate area of the basal plate and snap-frozen immediately. Calcified, necrotic, and seriously damaged areas were excluded. Next, every single biopsy was processed in two technical duplicates for DNA, mRNA, and protein isolation. Maternal demographics, obstetric, and neonatal outcomes were recorded.

### 2.3. DNA Isolation and Global DNA Methylation (5-mC) Assay

Total purified DNA was extracted from placental biopsies using Exgene Clinic SV Mini kit (GeneAll Biotechnology Co., Ltd., Seoul, Republic of Korea) following the manufacturer’s instructions. Briefly, after the addition of optimized lytic enzyme-containing buffer and Proteinase K, 20 mg of placental tissue were lysed at 56 °C. In these conditions, DNA binds to the silica membrane, while impurities pass through the membrane and are then discarded. Washes with a series of alcohol-containing buffers were performed to remove any traces of proteins, cellular debris, and salts. Finally, pure DNA was obtained with a low ionic strength buffer. DNA was measured spectrophotometrically, and the purity of the extract was based on the 260/280 O.D. ratio.

The percentage of global DNA methylation was calculated using the TheMethylFlash™ Global DNA Methylation (5-mC) ELISA Easy Kit (EpiGentek Group, Farmingdale, NY, USA) according to the manufacturer’s instructions. The methylated fraction of DNA was detected using capture and detection antibodies and then quantified colorimetrically by reading the absorbance at 450 nm in a microplate spectrophotometer. In this setting, the percentage of methylated DNA is proportional to the OD intensity measured. For each positive control, the concentration was plotted by the optical density to create a standard curve. The slope of the standard curve was determined using linear regression and was used to calculate the concentration of global 5-mC of each sample.

### 2.4. RNA Isolation and Real-Time PCR

Total RNA was isolated from frozen placental biopsies using TRI^®^ reagent (Sigma-Aldrich, Milan, Italy) according to the manufacturer’s instructions, and then treated with DNase I to remove genomic DNA contamination. Three micrograms of total RNA were reverse-transcribed using a random-hexamer approach (Fermentas Europe, St. Leon-Rot, Germany) and a RevertAid H Minus First Strand cDNA synthesis kit (Fermentas, Cat. No k1632, St. Leon-Rot, Germany). Gene expression levels of angiogenic factors VEGF (Hs0090055_m1), PlGF (Hs00182176_m1), and sFlt-1 (Hs01052961_m1) were determined by Real-Time PCR using specific TaqMan primers and probes following the manufacturer’s protocol (Life Technologies, Carlsbad, CA, USA, Cat. No 4331182). For relative quantification, PCR signals were compared between the groups after normalization using ribosomal 18S RNA expression as an internal reference (Life Technologies, Carlsbad, CA, USA, Cat. No 4333760F). In addition, GAPDH (Hs02786624_g1) was included as a supplementary reference gene to further improve normalization accuracy. Relative expression and fold change were calculated according to Livak and Schmittgen [27].

### 2.5. Enzyme-Linked Immunosorbent Assay (ELISA)

Total proteins were isolated from placental biopsies using 1× Radio Immuno-Precipitation Assay (RIPA) buffer supplemented with Protease Inhibitors. Quantitative measurement of VEGF, PlGF, and s-Flt-1 placental levels was determined using commercially available competitive ELISA kits (R&D System, Montebello Vicentino, Italy) according to the manufacturer’s instructions. ELISA’s optical density was determined at 450 nm.

### 2.6. Statistical Analysis

All parametric data are represented as mean  ±  standard error (SE), while non-parametric data are shown as median and range. Data normality was assessed prior to statistical analysis using the Shapiro–Wilk test. Comparison among groups was performed by analysis of variance. Bonferroni’s test was used for post hoc comparisons between two groups of parametric data, while Kruskal–Wallis and Mann–Whitney tests were used for non-parametric data. Categorical variables are presented as frequencies (percentages), and the comparison between different groups was done with the chi-square test. In addition, a multivariable regression analysis was performed, including maternal age as a covariate in order to control for its potential confounding effect on molecular data. Statistical tests were carried out using SPSS Version 29 software; the significance level was set at *p*  <  0.05.

## 3. Results

### 3.1. Clinical Features of the Study Population

The clinical characteristics of the study population are summarized in Table 1. Controls, ET, FET, and ED pregnancies were comparable for pre-pregnancy and term-pregnancy BMI, gestational weight gain, blood pressure, cesarean section rate, and neonatal APGAR scores. Uterine and umbilical artery Doppler measurements showed normal healthy waveforms across all groups (*p* > 0.05). As expected, maternal age at delivery was significantly higher in the ET (*p* = 0.002), FET (*p* = 0.001), and ED (*p* < 0.001) groups compared to controls (CTRL group). Maternal age was also significantly higher in the ED group than in both ET and FET pregnancies (*p* < 0.001). Gestational age at delivery was significantly lower in the ED (*p* = 0.002) and FET (*p* = 0.044) groups compared to controls. The percentage of women in their first pregnancy was significantly increased in the ET and FET groups relative to CTRL (*p* = 0.031 and *p* = 0.007, respectively). Furthermore, a significant reduction in neonatal birth weight was observed in the ET (*p* = 0.006), FET (*p* = 0.029), and ED (*p* = 0.018) groups compared to CTRL. However, placental weight and placental efficiency did not differ among groups (*p* > 0.05). Similarly, the distribution of neonatal sex (female/male) was comparable across all pregnancy types (*p* > 0.05).

### 3.2. Global Placental DNA Methylation in Spontaneous, Homologous, and Egg Donation IVF Pregnancies

**Figure 1:**
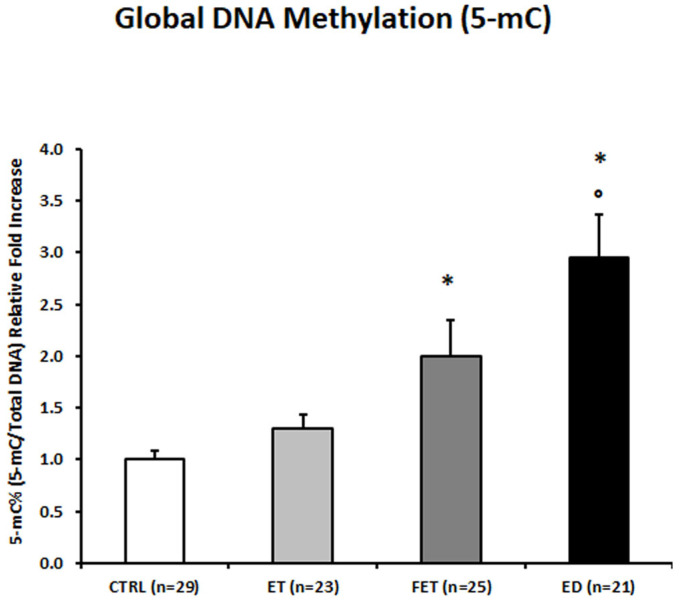
5-mC% placental global DNA methylation in CTRL, FET, ET, and ED pregnancies. Significant differences (*p* ≤ 0.05): * significant compared with CTRL and ° significant compared with ET.

Global placental DNA Methylation, expressed as the percentage of 5-methylcytosine (5mc), was significantly increased in ED placentae compared to both control (2.94-fold increase; *p* < 0.01) and ET placentae (2.27-fold increase; *p* = 0.006—Figure 1). FET placentae also showed a significant increase in global 5 mc levels compared to CTRL placentae (2-fold increase; *p* = 0.003—Figure 1).

Given the significant differences in maternal age among the study groups, an additional sub-analysis was performed by restricting the cohort to the only overlapping maternal age range common to all groups (35–40 years). Although the number of subjects in this subgroup was limited (particularly in the ED group), the distribution of global DNA methylation levels across ED, ET, FET, and CTRL placentae showed the same trend observed in the full cohort, with higher 5mc levels in ART-conceived pregnancies compared to spontaneous conceptions. However, due to the reduced sample size, these differences did not reach statistical significance (ET: *p* > 0.05, 1.2-fold increase; FET: *p* > 0.05, 1.7-fold increase; ED: *p* > 0.05, 2.6-fold increase) (Supplementary Figure S1).

In addition, a multivariable regression analysis, including maternal age as a covariate, was performed. After adjustment for maternal age, the association between ART conception and increased global placental DNA methylation remained significant, confirming that the observed differences are independent of maternal age.

### 3.3. Differential Effect of Fresh/Thawed Embryo Transfer on Placental Angiogenic Biomarkers

**Figure 2:**
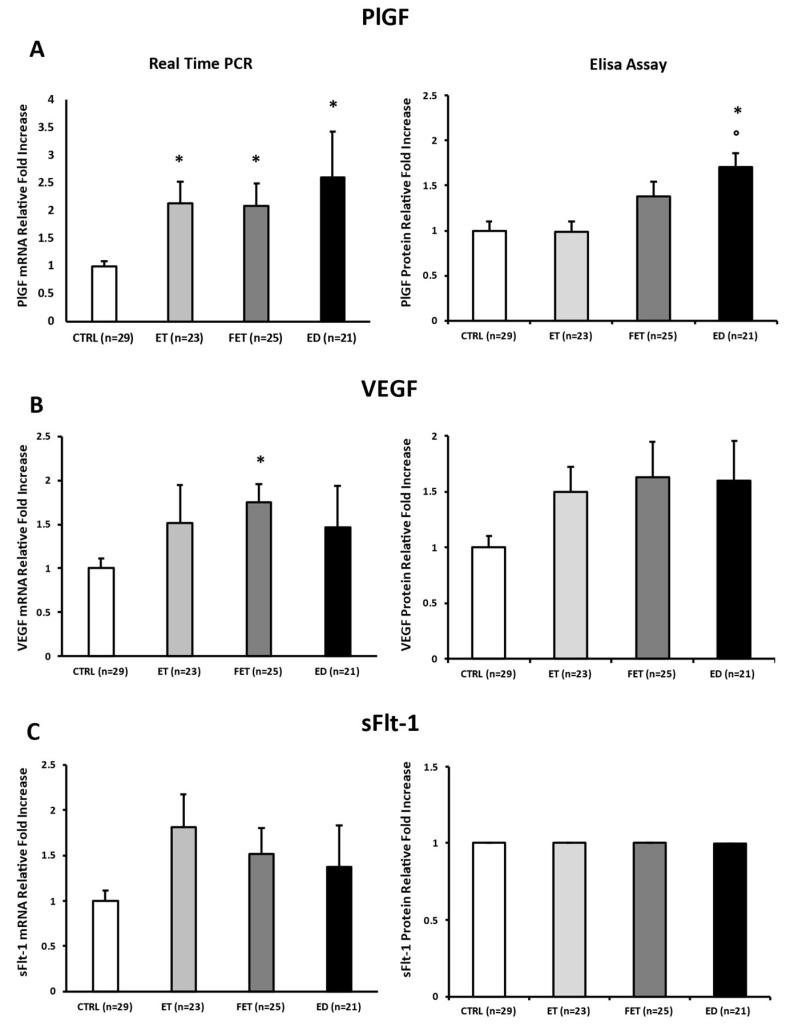
PlGF (**A**), VEGF (**B**), and sFlt-1 (**C**) gene and protein expression levels in CTRL, FET, ET, and ED pregnancies. Significant differences (*p* ≤ 0.05): * significant compared with CTRL and ° significant compared with ET.

Placental expression levels of pro-angiogenic PlGF were significantly upregulated at the mRNA level in ET, FET, and ED placentae relative to CTRL (ET: *p* = 0.001, 2.1-fold increase; FET: *p* = 0.005, 2.1-fold increase; ED: *p* = 0.017, 2.6-fold increase; Figure 2A, left panel). However, at protein levels, PlGF was significantly elevated only in ED placentae compared with both CTRL and ET placentae (*p* < 0.001, 1.7-fold increase; *p* < 0.001, 1.72-fold increase, respectively; Figure 2A, right panel).

VEGF mRNA expression was significantly increased in FET placentae compared with CTRL (*p* < 0.001; 1.74-fold increase; Figure 2B left panel), whereas protein levels did not significantly differ among groups, although ET, FET, and ED placentae showed a consistent upward trend (*p* > 0.05; Figure 2B right panel).

Finally, no significant differences were found in anti-angiogenic sFlt-1 at either mRNA and protein levels across all groups (*p* > 0.05; Figure 2C).

To further support the robustness of gene expression analyses, normalization was additionally performed using GAPDH as a supplementary reference gene, and the observed trends in VEGF, PlGF, and sFlt-1 expression were consistent with those obtained using 18S rRNA (Supplementary Figure S2).

Considering the significant differences in maternal age across the study groups, an additional analysis was performed, restricting the cohort to the only common maternal age range (35–40 years). Despite the limited number of subjects in this subgroup, particularly in the ED group, the expression patterns of PlGF (gene—ET: *p* > 0.05, 1.8-fold increase; FET: *p* > 0.05, 1.4-fold increase; ED: *p* < 0.01, 4.2-fold increase; Supplementary Figure S3A, left panel. Protein—ET: *p* > 0.05, 1-fold increase; FET: *p* > 0.05, 1.1-fold increase; ED: *p* > 0.05, 1.5-fold increase; Supplementary Figure S3A, right panel), VEGF (gene—ET: *p* > 0.05, 2.1-fold increase; FET: *p* > 0.05, 2.2-fold increase; ED: *p* > 0.05, 2.9-fold increase; Supplementary Figure S3B, left panel. Protein—ET: *p* > 0.05, 1.3-fold increase; FET: *p* > 0.05, 1.2-fold increase; ED: *p* > 0.05, 1.5-fold increase; Supplementary Figure S3B, right panel) and sFlt-1 (gene—ET: *p* > 0.05, 1.8-fold increase; FET: *p* > 0.05, 2.2-fold increase; ED: *p* > 0.05, 3.4-fold increase; Supplementary Figure S3C, left panel. Protein—ET: *p* > 0.05, 1-fold increase; FET: *p* > 0.05, 1-fold increase; ED: *p* > 0.05, 1-fold increase; Supplementary Figure S3C, right panel) remained consistent with those observed in the overall cohort, with a general trend toward higher pro-angiogenic factor expression in ART-conceived pregnancies compared to spontaneous conceptions. However, these differences did not reach statistical significance due to reduced statistical power (Supplementary Figure S3).

In addition, a multivariable regression analysis including maternal age as a covariate was performed. After adjustment for maternal age, the differences between groups remained consistent with the primary analysis, confirming that the observed trends are not driven by maternal age.

## 4. Discussion

In the present study, we observed that placentae from physiological pregnancies conceived via IVF with homologous single vitrified-thawed blastocyst transfer or egg donation exhibit a significant increase in global DNA methylation compared to those from spontaneous conception. These epigenetic differences were accompanied by increased expression of key pro-angiogenic factors PlGF and VEGF. Indeed, our data suggest that a different epigenetic regulation of placental vascular development is associated with IVF itself and varies according to procedural aspects, such as embryo freezing/thawing and/or the use of donor oocytes. Importantly, the expression of the anti-angiogenic factor sFlt-1 remained unchanged, indicating a selective modulation of placental pro-angiogenic signaling pathways, rather than a generalized angiogenic shift.

Placentae from FET and ED pregnancies exhibited a hypermethylated profile compared to those from spontaneous conceptions. These findings are consistent with previous reports indicating that IVF can influence epigenetic programming in both embryonic and placental tissues. Alterations in global DNA methylation, including both hypermethylation and hypomethylation, have been previously observed in placentas from IVF pregnancies, likely reflecting epigenetic perturbations associated with ovarian stimulation, embryo culture, and transfer protocols [18,28,29]. Our findings, however, delineate more precisely the specific IVF procedures with the strongest epigenetic impact: a common feature of both FET and ED is the use of vitrified oocytes or embryos. Vitrification is a process widely associated with hypermethylation [30], involving extreme osmotic shifts and exposure to high concentrations of cryoprotectants, followed by rapid freezing and warming. These stressors can interfere with the activity of DNA methyltransferases and chromatin remodeling machinery during critical windows of epigenetic reprogramming. Recent data suggest that such disruption may lead to persistent epigenetic alterations, particularly in genomic regions important for placental development and stability, such as repetitive elements and imprinted loci [31].

Our findings support the hypothesis that the hypermethylation observed in FET placentae is more likely attributable to the vitrification/thawing process than to the hormonal environment at the time of embryo transfer, as it is absent in ET pregnancies obtained with fresh embryos, despite their more adverse hormonal milieu. Although FET cycles occur under more physiological endocrine conditions than fresh transfers, the epigenetic impact of the cryopreservation appears to be a dominant factor. This is further corroborated by evidence showing that vitrification-induced stress can lead to Reactive Oxygen Species (ROS) accumulation, DNA damage, and disruption of both epigenetic modifications and transcriptome profiles [32]. Importantly, all IVF subgroups in our study—ET, FET, and ED—received the same regimen of transvaginal progesterone supplementation (600 mg/day) during the luteal phase and up to 9 weeks of gestation. Therefore, progesterone exposure was uniform across groups and cannot account for the distinct hypermethylation profiles or the differential expression of PlGF and VEGF observed specifically in FET and ED placentae. Available evidence also indicates that standard luteal-phase progesterone support does not induce global DNA hypermethylation nor selectively modulate placental angiogenic gene expression [33,34]; its primary effects during implantation are immunomodulatory and decidual, rather than epigenetic [23,35,36,37]. If progesterone were driving the observed molecular differences, similar methylation and angiogenic patterns would be expected across all ART groups. The persistence of these alterations at term—long after progesterone supplementation is discontinued—further supports the conclusion that they arise from procedure-specific factors such as vitrification and donor oocyte origin rather than progesterone exposure itself.

Similarly, ED pregnancies represent a uniquely complex model, influenced not only by embryo vitrification but also by the biological and epigenetic variability of donor oocytes. In our cohort, ED embryos underwent cryopreservation, thereby combining two distinct sources of potential epigenetic disturbance: the non-autologous (donor-derived) genetic/epigenetic material and the cryopreservation process. The donor oocyte’s intrinsic epigenetic state, its potential mismatch with the recipient’s uterine environment, and its exposure to ex vivo manipulation may all contribute to the distinct methylation profiles observed in ED placentae.

The significant increase in global DNA methylation in FET and ED placentae observed in our study was accompanied by upregulated PlGF and VEGF mRNA expression, suggesting a potential link between epigenetic modifications and altered regulation of key angiogenic pathways. Interestingly, despite these molecular changes, all placentae remained physiological, indicating that such alterations do not necessarily translate into pathological outcomes as previously described [33,34]. At the protein level, only PlGF was significantly elevated in ED placentae, while VEGF levels remained comparable among groups. These differences between mRNA and protein expression point to the existence of post-transcriptional compensatory mechanisms that may buffer or fine-tune gene expression to preserve placental function despite upstream molecular alterations.

Interestingly, sFlt-1 expression remained unchanged among groups. This finding is particularly important as it suggests that the angiogenic pathways may be more resistant to IVF-associated epigenetic perturbations or may be governed by distinct regulatory mechanisms not influenced by global methylation changes. These observations are consistent with those of Woo, who found no significant differences in maternal serum sFlt-1 and PlGF levels between ET and FET pregnancies [4], further supporting the notion that angiogenic balance can be maintained in IVF pregnancies through robust regulatory mechanisms.

Although maternal age differed significantly among the groups, particularly in the ED cohort, our additional age-restricted analysis suggests that age alone is unlikely to fully account for the epigenetic and angiogenic differences observed in ART-derived placentae. Indeed, when the comparison was limited to the only overlapping maternal age range shared by all groups (35–40 years), the same biological pattern—characterized by higher global DNA methylation and a tendency toward increased pro-angiogenic signaling in ART pregnancies—was still evident, despite the markedly reduced sample size. While statistical significance was not achieved in this restricted analysis, the persistence of the same directional trend strongly supports the interpretation that the observed molecular alterations are primarily driven by IVF-related procedural factors rather than maternal age per se. This observation strengthens the interpretation that IVF-related procedures exert an independent influence on placental epigenetic programming and angiogenic regulation, beyond the contribution of advanced maternal age, which is itself a recognized but not exclusive determinant of placental and fetal adaptations.

Importantly, this apparent “contradiction” between hypermethylation and increased pro-angiogenic gene expression is biologically plausible. Global DNA methylation does not necessarily predict gene-specific methylation states, particularly in promoters and enhancers controlling PlGF and VEGF transcription. Global hypermethylation often reflects methylation of repetitive or non-regulatory regions and can coexist with permissive chromatin states at key regulatory loci [38,39,40]. Thus, the hypermethylated signature observed in IVF placentae likely reflects widespread epigenomic remodeling, while angiogenic genes remain selectively protected or transcriptionally activated. Increasing evidence indicates that ART does not trigger uniform gene repression through hypermethylation but rather induces gene-specific epigenetic remodeling [18,41,42]. In this context, the upregulation of PlGF and VEGF may represent a compensatory or adaptive response aimed at sustaining villous vascular development in the face of early developmental or procedural stressors—including embryo culture conditions, vitrification, and the non-autologous origin of donor oocytes. The stability of sFlt-1 expression further supports the idea that angiogenic pathways are governed by distinct regulatory mechanisms that remain resilient to global methylation shifts.

It is well known that IVF pregnancies exhibit a distinct risk of adverse perinatal outcomes, mainly linked to patients’ characteristics (e.g., relatively advanced age) and potentially influenced by embryo manipulation and culture conditions. It is generally reported that FET pregnancies are associated with a higher incidence of large-for-gestational-age (LGA) neonates [43,44,45,46], whereas fresh ET is more often linked to small-for-gestational-age (SGA) outcomes [46,47]. However, we observed a significantly lower neonatal birth weight in all IVF groups compared to the CTRL group, with no significant differences observed among the ET, FET, and ED subgroups. This is in contrast with the expected pattern and suggests that, in our population, the reduction in neonatal size is not dependent on the type of embryo transfer but may instead reflect a broader effect of IVF itself. Subtle placental adaptations or epigenetic modifications induced by in vitro embryo culture conditions may influence fetal growth trajectories independently of transfer modality. These may involve global DNA methylation patterns and the expression changes in angiogenic factors such as VEGF and PlGF. Such findings support the hypothesis that IVF-related epigenetic remodeling may influence placental vascular development and nutrient transport capacity, thereby modulating fetal growth even in the absence of overt pathology.

Despite the observed reductions, all neonates had birth weights within the normal physiological range, and there were no cases of low birth weight (1500–2500 g), very low birth weight (<1500 g), or preterm birth (<37 weeks of gestation). These findings are consistent with previous studies: Sekhon analyzed 202 single embryo transfers from egg donation and found no significant difference in birth weight between fresh and vitrified euploid embryos. Similarly, when comparing 2760 autologous vitrified euploid embryo transfers, no difference in birth weight was observed [48]. Another retrospective study involving 516 cycles undergoing either FET or fresh embryo transfer also reported no significant differences in neonatal birth weights [49]. Despite the lower average birth weight observed in our IVF cohorts, neonatal outcomes, including placental weight and efficiency, were comparable across all groups, suggesting that immediate neonatal health and adaptation were not adversely affected.

Importantly, our results also have clinical implications. Although all pregnancies in our cohort remained uncomplicated, the presence of distinct hypermethylation patterns and selective upregulation of pro-angiogenic markers suggests that certain IVF subgroups may undergo unique placental adaptations, not fully captured by standard obstetric assessments. Pregnancies conceived through embryo vitrification or using donor oocytes may, therefore, benefit from more tailored obstetric surveillance, particularly focusing on placental function and fetal growth. Targeted monitoring strategies—such as serial third-trimester growth ultrasounds, Doppler velocimetry of the uterine and umbilical arteries and evaluation of angiogenic biomarkers—could support earlier identification of subtle placental dysfunction, especially in women with additional risk factors (e.g., advanced maternal age, obesity, chronic conditions). Furthermore, the altered expression of PlGF and VEGF provides a biological rationale for future evaluation of first- or second-trimester angiogenic profiling as an adjunct tool in ART pregnancies. Although sFlt-1 remained stable, preservation of angiogenic balance might not fully eliminate the possibility that the placenta responds differently to maternal stressors. This may be particularly relevant in FET and ED pregnancies, where both vitrification and donor oocyte–related variability could potentially affect early placental programming.

Overall, these findings support the concept that IVF pregnancies represent a distinct clinical entity characterized by selective epigenetic remodeling, compensatory activation of angiogenic pathways, and preserved placental function. Personalized obstetric management may help optimize maternal–fetal outcomes, particularly in those IVF subgroups most exposed to epigenetic perturbations.

## 5. Limitations

Some limitations of this study should be acknowledged. While global DNA methylation provides a broad overview of epigenetic alterations, it does not capture gene-specific changes. Genome-wide or targeted methylation profiling would allow for the identification of Differentially Methylated Regions (DMRs), particularly in regulatory elements relevant to placental development and angiogenesis. Functional studies are required to clarify the causal relationship between altered DNA methylation and the expression of angiogenic genes, as well as the downstream effects on placental vascularization and nutrient transport.

Another limitation is the absence of a comparison group of ED pregnancies derived from fresh embryo transfers, which would have allowed us to better distinguish the respective contributions of oocyte origin and embryo vitrification to the observed methylation changes. Including such a group in future studies would help to determine whether the hypermethylation pattern identified in ED placentae primarily reflects the cryopreservation process or the intrinsic donor oocyte epigenetic features.

In addition, although we performed a sub-analysis restricted to the only overlapping maternal age range among all groups (35–40 years), the limited number of cases in all subgroups within this interval reduced the statistical power of the comparison. While the observed trends remained consistent with those of the overall cohort, this age-restricted analysis did not reach statistical significance, and larger, age-matched cohorts will be required in future studies to more robustly disentangle the relative contributions of maternal age and ART-related procedures to placental epigenetic remodeling.

Finally, longitudinal follow-up of ART-conceived offspring is essential to determine whether these placental epigenetic changes have lasting effects on neonatal, childhood, or even adult health. Establishing such connections would not only enhance our understanding of ART-related risks but could also inform strategies for early monitoring and intervention.

## 6. Conclusions

In conclusion, the hypermethylation observed in FET and ED placentae may serve as a compensatory or protective mechanism, potentially aimed at silencing transposable elements and stabilizing genomic regions susceptible to IVF-induced stress. In ED pregnancies, the hypermethylation pattern likely reflects the combined influence of embryo vitrification and donor oocyte origin. This epigenetic “buffering” could promote the expression of angiogenic factors as part of an adaptive response to support placental vascular development and maintain fetal viability, particularly under suboptimal implantation conditions or in the context of donor oocyte-derived epigenomes. Such a mechanism may help explain why placental efficiency and neonatal outcomes remain largely preserved, despite the reduced birth weight reported in IVF-conceived infants. Further research is needed to elucidate the mechanisms underlying these changes and to translate these insights into improved ART practices.

## Figures and Tables

**Table 1:** Clinical features of the study population. Values are expressed as median ± range and percentage. Significant differences (*p* ≤ 0.05): * differences indicating a significant effect compared with CTRL; ° differences indicating a significant effect compared with ET pregnancies and § differences indicating a significant effect compared with FET pregnancies.

	CTRL (n = 29)	ET (n = 23)	FET (n = 25)	ED (n = 21)	*p*-Value
**First pregnancy (%)**	31.0 (n = 9)	* 56.5 (n = 13)	* 64.0 (n = 16)	47.6 (n = 10)	ET vs. CTRL *p* = 0.031 FET vs. CTRL *p* = 0.007
**Pre-pregnancy BMI (kg/m^2^)**	21.1 (17.7–34.7)	21.4 (17.9–28.0)	20.4 (17.6–26.7)	21.7 (18.6–28.6)	*p* > 0.05
**FSH (mlU/mL)**		7.0 (4.1–12.0)	7.1 (2.8–9.1)		*p* > 0.05
**AMH (ng/mL)**		2.2 (1.2–6.7)	3.9 (1.7–19.4)		*p* > 0.05
**AFC**		14.5 (8.0–30.0)	18.0 (10.0–35.0)		*p* > 0.05
**Normal uterine artery Doppler (%)**	100	100	100	100	*p* > 0.05
**Normal umbilical artery Doppler (%)**	100	100	100	100	*p* > 0.05
**Maternal age at delivery (years)**	34.0 (25–39)	36.0 * (25–41)	37.5 * (32–42)	44.0 * § ° (37–51)	ET vs. CTRL *p* = 0.022 FET vs. CTRL *p* = 0.001 ED vs. CTRL, ET and FET *p* < 0.001
**Gestational age at delivery (weeks)**	40.0 (37.3–42.4)	39.3 (34.4–41.6)	39.4 (34.6–41.7)	38.9 * § (36.4–41.3)	ED vs. CTRL *p* = 0.002 ED vs. FET *p* = 0.044
**Gestational weight gain (kg)**	13.5 (10.0–18.5)	12.0 (3.0–16.0)	13.0 (6.0–25.0)	11.0 (−3.0–15.0)	*p* > 0.05
**Delivery BMI (kg/m^2^)**	25.9 (20.3–40.0)	26.0 (22.7–33.7)	25.9 (21.2–33.4)	25.5 (18.1–36.4)	*p* > 0.05
**Systolic blood pressure (mm Hg)**	120.0 (100.0–140.0)	115.0 (90.0–141.0)	121.0 (100.0–150.0)	120.0 (103.0–137.0)	*p* > 0.05
**Diastolic blood pressure (mm Hg)**	73.0 (55.0–92.0)	70.0 (55.0–92.0)	75.0 (55.0–92.0)	72.0 (60.0–90.0)	*p* > 0.05
**Labor (%)**	82.1 (n = 23)	68.2 (n = 15)	68.0 (n = 17)	52.4 (n = 11)	*p* > 0.05
**Cesarean section (%)**	31 (n = 9)	30.4 (n = 7)	52.0 (n = 13)	57.1 (n = 12)	*p* > 0.05
**Birth weight (g)**	3665.0 (3310–4030)	3145.0 * (2140–4650)	3080.0 * (2380–3500)	3260 * (2690–3760)	ET vs. CTRL *p* = 0.006 FET vs. CTRL *p* = 0.029 ED vs. CTRL *p* = 0.018
**Placental weight (g)**	640.0 (470–870.0)	550.0 (415.0–750.0)	550.0 (360.0–700.0)	560.0 (450.0–740.0)	*p* > 0.05
**Placental efficiency (F/P ratio)**	5.6 (4.7–7.0)	5.8 (4.3–7.7)	5.7 (3.5–7.9)	6.0 (4.1–7.4)	*p* > 0.05
**Fetal female sex (%)**	37.9 (n = 11)	43.5 (n = 10)	52.0 (n = 13)	47.6 (n = 10)	*p* > 0.05
**APGAR 5” ≤ 7 (%)**	0	0	4 (n = 1)	4.76 (n = 1)	*p* > 0.05

## Data Availability

The datasets used and/or analyzed during the current study are available from the corresponding author on reasonable request.

## Supplementary Materials

The following supporting information can be downloaded at: https://www.mdpi.com/article/10.3390/life15121882/s1. Figure S1: 5-mC% placental global DNA methylation in CTRL, ET, FET, and ED pregnancies within the overlapping maternal age range (35–40 years). Figure S2: PlGF, VEGF, and sFlt-1 placental gene expression normalized using GAPDH as a reference gene. Significant differences (*p* ≤ 0.05): * significant compared with CTRL. Figure S3: PlGF (A) VEGF (B) and sFlt-1 (C) gene and protein expression levels in CTRL, FET, ET and ED pregnancies within the overlapping maternal age range (35–40 years). Significant differences (*p* ≤ 0.05): * significant compared with CTRL; ° significant compared with ET and § significant compared with FET.

## Abbreviations

The following abbreviations are used in this manuscript:

5-mC	5-methylcytosine
AFC	Antral Follicle Count
AMH	Anti-Müllerian Hormone
ART	Assisted Reproductive Technology
BMI	Body Mass Index
COS	Controlled Ovarian Stimulation
CTRL	Control
ED	Egg Donation
ELISA	Enzyme-Linked Immunosorbent Assay
ET	Fresh Embryo Transfer
FET	Frozen–Thawed Embryo Transfer
FSH	Follicle-Stimulating Hormone
ICSI	Intra-Cytoplasmic Sperm Injection
LGA	Large-for-Gestational-Age
PCR	Polymerase Chain Reaction
PE	Preeclampsia
PI	Pulsatility Index
PlGF	Placental Growth Factor
ROS	Reactive Oxygen Species
SGA	Small-for-Gestational-Age
sFlt-1	Soluble Fms-Like Tyrosine Kinase-1
SE	Standard Error
VEGF	Vascular Endothelial Growth Factor
